# Repartitioning of glycerol between levitated and surrounding deposited glycerol/NaNO_3_/H_2_O droplets

**DOI:** 10.1098/rsos.170819

**Published:** 2018-01-03

**Authors:** Xiaoyan Gao, Chen Cai, Jiabi Ma, Yunhong Zhang

**Affiliations:** The Institute of Chemical Physics, Key Laboratory of Cluster Science, School of Chemistry and Chemical Engineering, Beijing Institute of Technology, Beijing 100081, People's Republic of China

**Keywords:** repartitioning, aerosol, optical tweezers, glycerol/NaNO_3_/H_2_O, SVOCs

## Abstract

Repartitioning of semi-volatile organic compounds (SVOCs) between particles is an important process to understand the particle growth and shrinkage in the atmosphere environment. Here, by using optical tweezers coupled with cavity-enhanced Raman spectroscopy, we report the repartitioning of glycerol between a levitated glycerol/NaNO_3_/H_2_O droplet and surrounding glycerol/NaNO_3_/H_2_O droplets deposited on the inner wall of a chamber with different organic to inorganic molar ratios (OIRs). For the high OIR with 3 : 1, no NaNO_3_ crystallization occurs both for levitated and deposited droplets in the whole relative humidity (RH) range, the radius of the levitated droplet decreases slowly due to the evaporation of glycerol from the levitated droplet at constant RHs. The levitated droplets radii with OIR of 1 : 1 and 1 : 3 increase with constant RHs that are lower than 45.3% and 55.7%, respectively, indicating that the repartitioning of glycerol occurs. The reason is that NaNO_3_ in the deposited droplets is crystallized when RH is lower than 45.3% for 1 : 1 or 55.7% for 1 : 3. So the vapour pressure of glycerol at the surface of deposited droplets is higher than that of the levitated droplet which always remains as liquid droplet without NaNO_3_ crystallization, resulting in the transfer of glycerol from the deposited ones to the levitated one. The process of the glycerol repartitioning we discussed herein is a useful model to interpret the repartitioning of SVOCs between the externally mixed particles with different phase states.

## 1. Introduction

Particulate matter in Earth's atmosphere, also known as atmospheric aerosols, plays critical roles in air quality, climate and atmospheric chemistry [1,2]. Both particle formation and particulate evolution processes in Beijing haze are not clear because of complex sources and thus complex gas–particle transformation and particle ageing processes. The serious air pollution caused by fine particles in Beijing affects the accuracy of weather forecasts [3].

New particles formation and their subsequent growth, which are frequently observed in the atmosphere [4], are important sources of atmospheric aerosols, and can lead to increase of aerosol number and mass concentrations. In the past decade, the formation and growth of new particles have attracted much interest owing to its potential influence on the climate by interacting effectively with solar radiation or participating in cloud formation processes [5–7]. Numerous studies have shown that the relative low vapour pressures of organic compounds such as polyalcohols and dicarboxylic acids, which are also known as semi-volatile organic compounds (SVOCs), were found to be responsible for up to 90% of the observed growth of newly formed particles [8,9]. SVOCs as major components of atmospheric aerosols are generated through the oxidative ageing of anthropogenic and biogenic volatile organic compounds and further lead to the formation of secondary organic aerosol [10–13]. Previous literature indicates that the processes of new particles growth are mainly due to the condensation of SVOCs in the gas phase [7,14,15]. On the other hand, the SVOCs aerosol particles usually start as an external mixture with many populations from varied local sources, and then may become internally mixed population via gas phase exchange of SVOCs between particles [16,17]. Understanding particle mixing states and repartitioning of SVOCs between particles is important not only for source apportionment in urban environments but also for modelling their impact on climate and air quality [18]. Notably, recent studies have reported a reversal process leading to particle shrinkage, which can also occur due to the evaporation of condensed SVOCs [19–23]. Enhanced atmospheric dilution, high ambient temperature and low relative humidity (RH), which result in the increasing of precursor vapour concentrations and favouring the evaporation of SVOCs from the particulate phase to the gas phase, can lead to particle shrinkage. Moreover, the processes of particle growth and shrinkage are reversible and SVOCs can repartition between the gas and particle phases as well as between particles when condition changes. In order to get a clear understanding of the processes governing particles growth and shrinkage in the atmosphere, it is quite necessary to study the repartitioning of SVOCs between gas and particles, and between externally mixed particles with different conditions in the atmospheric environment.

A single-beam gradient force optical trapping method, i.e. optical tweezers, can capture and manipulate single particle, 2–10 µm in radius. Since 2004, optical tweezers coupled with Raman spectroscopy has been a useful tool to investigate the suspended droplets to simulate the atmospheric environment and probe evolving particle sizes (with nanometre accuracy), compositions, phases and mixing states [24–27]. Thus, it can be used to investigate the thermodynamic properties of aerosol and the kinetics of particle transformation and water transport in the gel or glassy aerosols [26–35]. For example, using the optical tweezers, Hanford *et al*. [28] studied the hygroscopic proterties of glutaric acid/ammonium sulfate droplet and glutaric acid/sodium chloride droplet; Cai *et al*. [27] detemined slow water transport in MgSO_4_ droplets at gel-forming RHs, and the hygroscopicity as well as the vapour pressures of SVOC droplets including dicarboxylic acids and polyalcohols [29,30]; Dennis-Smither *et al*. [31] investigated volatility and oxidative ageing of maleic acid aerosol droplets; Tong *et al.* [34] determined the timescales for the mass transfer of water in sucrose and sucrose/sodium chloride droplets at low RHs; Stewart *et al*. [35] investigated the phase separation RHs and morphologies of polyethylene glycol/ammonium sulfate and C6-diacids/ammonium sulfate droplets.

The reported studies show that optical tweezers is a good method to study partitioning of SVOCs between gas and particle phases [29–32]. Our investigation herein indicates that it is also a unique method to detect the repartitioning of SVOCs between the trapped droplet and its surrounding deposited particles on the inner wall of the chamber, which have different phase states. The crystal nucleation in optical levitated droplet can be deemed as homogeneous process, while its surrounding deposited particles experience heterogeneous nucleation with decreasing RH. In this case, phase state of the trapped droplet can be controlled to be different from the deposited particles even at the same RH. Thus it is a good model to study SVOCs transfer between externally mixed particles with different conditions.

Actually, aerosol particles with different compositions can give significant impacts on Earth climate and human health (including the respiratory system, the cardiovascular system, the infectious diseases and even cancer diseases) [36–38]. Field measurements have shown that atmospheric aerosols always include both inorganic and organic compounds [39], and the molar ratios of organic to inorganic species (referred to as OIRs) are various depending on the location, season and other environmental conditions [40–42]. It is necessary to study the mixed organic/inorganic particles with different molar ratios using a new method for making different phenomena clear. As one important class of SVOCs, polyalcohols, such as glycerol, is widely used in industry and other fields [43]. Although glycerol is a water-soluble SVOC found in atmospheric aerosols, it does not show efflorescence RH over its entire RH range [44]. In addition, NaNO_3_ is one of the most common inorganic constituents in atmospheric aerosols, which is the product of the reactions of fresh sea salt aerosols (primary aerosols) with nitrogen oxides, such as HNO_3_, N_2_O_5_, NO_2_ and NO_3_ [45,46]. Glycerol is an organic compound with three OH groups that could interact with Na^+^ and ${\mathrm{NO}}_{3}{}^{-}$ ions. The hydroscopic behaviour of the mixture of glycerol and NaNO_3_ with different OIRs has been studied [47,48]. Yu *et al*. [47] using micro-Raman spectroscopy found that the efflorescence RH of NaNO_3_ decreased with increasing glycerol compared with that of pure NaNO_3_ (47–53%); when the OIR of mixed aqueous droplet increased to 2 : 1, the droplet does not form crystal even at very low RH (2%) for an extended period of time. Ren *et al*. [48] also found that efflorescence RH of glycerol/NaNO_3_ droplets is lower than that of pure NaNO_3_ (48.9–63.7%) by Fourier transform infrared attenuated total reflection technique. The reason they concluded was that the glycerol molecules suppress the formation of contact ion pairs between Na^+^ and $\text{N}{{\text{O}}_{3}}^{-}$, which changes the efflorescence of NaNO_3_ in the mixed glycerol and NaNO_3_ droplets.

Previous studies mainly focus on the effect of glycerol on NaNO_3_ hygroscopicity. Little attention has been paid to the process of glycerol transfer between different phase states of glycerol/NaNO_3_/H_2_O. In this work, for the first time, optical tweezers coupled with cavity-enhanced Raman spectroscopic was employed to study the repartitioning of glycerol between a levitated glycerol/NaNO_3_/H_2_O droplet and its surrounding deposited glycerol/NaNO_3_/H_2_O droplets with different OIRs of 3 : 1, 1 : 1 and 1 : 3 when the RH changes.

## 2. Experiments

Glycerol (analytical grade, 99.0%, Beijing Chemical Reagents Company) and NaNO_3_ (analytical grade, 99.0%, Beijing Chemical Reagents Company) were dissolved by triply deionized water to get mixed glycerol/NaNO_3_ aqueous solution. All chemicals were used directly without further treatment. The glycerol to NaNO_3_ molar ratios in the mixed glycerol/NaNO_3_ aqueous solution prepared are 3 : 1, 1 : 1 and 1 : 3, respectively.

**Figure 1.**
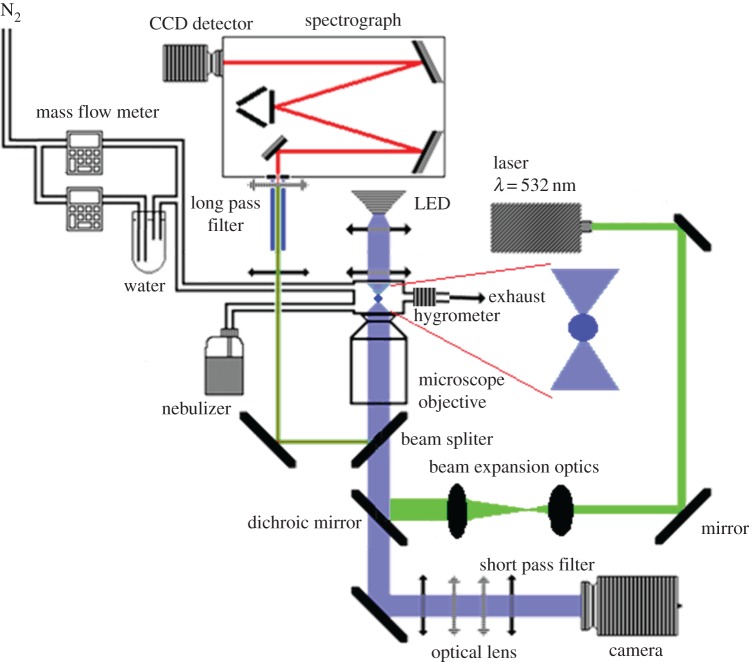
Schematic diagram of experimental set-up used for single droplet optical tweezing.

The aerosol optical tweezers technique for measuring the single aqueous particles has been comprehensively described in previous publications [26,29,49–52]. Figure 1 shows a schematic diagram of the single-gradient force optical trap used in this study. The light beam from a diode-pumped solid-state laser (Opus 4 W + mpc6000 power supply, 532 nm) first passes through two sets of beam expansion optics, reflected from a dichroic mirror and then from a 50 : 50 beam splitter, the optical trap was formed by focusing light through a 100× oil immersion microscope objective (numerical aperture of 1.25). The laser power used here was 200 mW. A dispersed aerosol mist was generated using a portable ultrasonic nebulizer (MY-520, Shenzhen) and introduced into the laser trapping sample chamber where a single particle was confined and enlarged by coalescence with further particles in the plume. Particles containing NaNO_3_ and glycerol as the solute were produced by nebulizing an aqueous solution of glycerol/NaNO_3_ as described above. A single particle of 2–10 µm in radius was captured and held near the beam focus at a height of 40 µm above a thin glass coverslip mounted above the microscope objective. The chamber (approx. 7 cm^3^) has three ports which can allow the introduction of the aerosol flow, a humidified stream of nitrogen gas and serve as outlets for the gas and excess aerosol flow. After the particle was captured, the introduction of the aerosol flow port was subsequently sealed by high vacuum grease (Dow Corning Company) and a humidified nitrogen flow was introduced to equilibrate the chamber and particle at a known RH. By adjusting the balance between flows of dry nitrogen gas and humidified nitrogen gas through a bubbler containing deionized water using needle valves, the RH in the cell can be regulated. The mass flow rate of each flow was controlled by different needle valves and displayed by mass flow meters (Alicat Scientific). The RH meter CENTER 313 was used to monitor the RH in the gas flow. The total mixed flow introduced into the cell was kept constant at 200 cm^3^ min^−1^.

A blue LED centred at 455 nm provides illumination for bright-field imaging of the trapped particles, and the light reflected from the beam splitter and dichroic mirror was projected onto a camera (Watec, 1/3 in., model 231S2) after removing scattered laser light with a short pass filter. This could collect images from the underside of the particle, referred to as in-plane imaging. The range of the laser and LED illumination wavelengths were removed by appropriate notch and long pass filters (around 514.5 nm) from the back-scattered Raman signal from the particle. The Raman scatter was focused onto the entrance slit of a spectrograph instrument (Omni-λ 5006) with a 1200 grooves nm^−1^ grating and a CCD (iDus DV420A-BV) array of 1024 × 256 pixels. The changing spectra of the particle were monitored in real time with a time-resolution of 1 s. Cavity-enhanced Raman spectra (CERS) were recorded by collecting the inelastically back-scattered light from a trapped particle and acquiring the wavelength-resolved spectrum using a spectrograph and CCD. The CERS fingerprint consisted of spontaneous and stimulated Raman scattering components, providing information on the composition, structure, refractive index and the size of trapped particles [26]. The spontaneous Raman scatter broad Stokes bands shifted from the incident radiation at 532 nm, was used to probe composition by assignment to the distinct vibrational modes of species within the particle. The stimulated Raman scatter arises from the capacity of the trapped particle to act as an optical cavity and leads to the formation of sharp peaks in the Raman spectra at wavelengths commensurate with whispering gallery modes (WGMs). The size and refractive index of the particle at each moment in time were determined in real time from the wavelength position of CERS in the particle, by use of the proprietary Biral and University of Bristol sizing software (LARA) incorporating the algorithms of Preston & Reid [53].

## 3. Results and discussion

### 3.1. Repartitioning of glycerol with different OIRs

**Figure 2.**
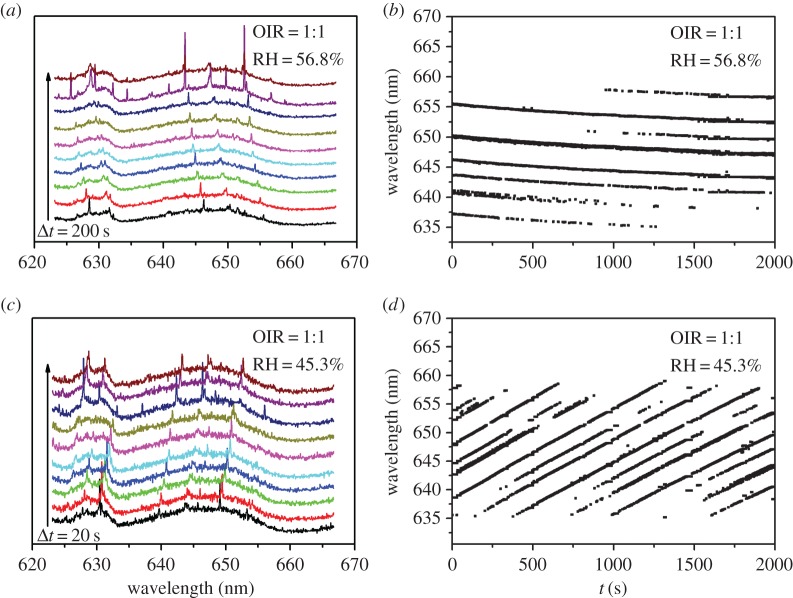
(*a*,*c*) CERS of glycerol/NaNO_3_/H_2_O droplet with OIR of 1 : 1 at 56.8% RH and 45.3% RH, respectively. (*b,d*) The time evolution of the wavelengths of the WGM resonant modes on the OH band for the glycerol/NaNO_3_/H_2_O droplet with OIR of 1 : 1 at 56.8% RH and 45.3% RH, respectively.

The aqueous droplets of glycerol/NaNO_3_/H_2_O with different OIRs were optically trapped by optical tweezers. The typical CERS examples of an aqueous glycerol/NaNO_3_/H_2_O droplet, in particular the varying wavelengths of the WGMs, is shown in figure 2. Spontaneous Raman scatter arises from excitation of the –OH stretching vibrations of water and is observed as a broad band extending from approximately 635 to 660 nm, a Stokes shift of between 3050 and 3650 cm^−1^ [28]. For the glycerol/NaNO_3_/H_2_O droplet with OIR of 1 : 1, the Raman peaks are shifted to the lower wavelength during acquisition time when the RH is 56.8%, and the corresponding results are shown in figure 2*a*,*b*. Whereas the peaks shift to higher wavelength when the RH is 45.3%, as shown in figure 2*c*,*d*. From the peak position of CERS, the radii of droplets can be calculated according to the previous literature [53].

**Figure 3.**
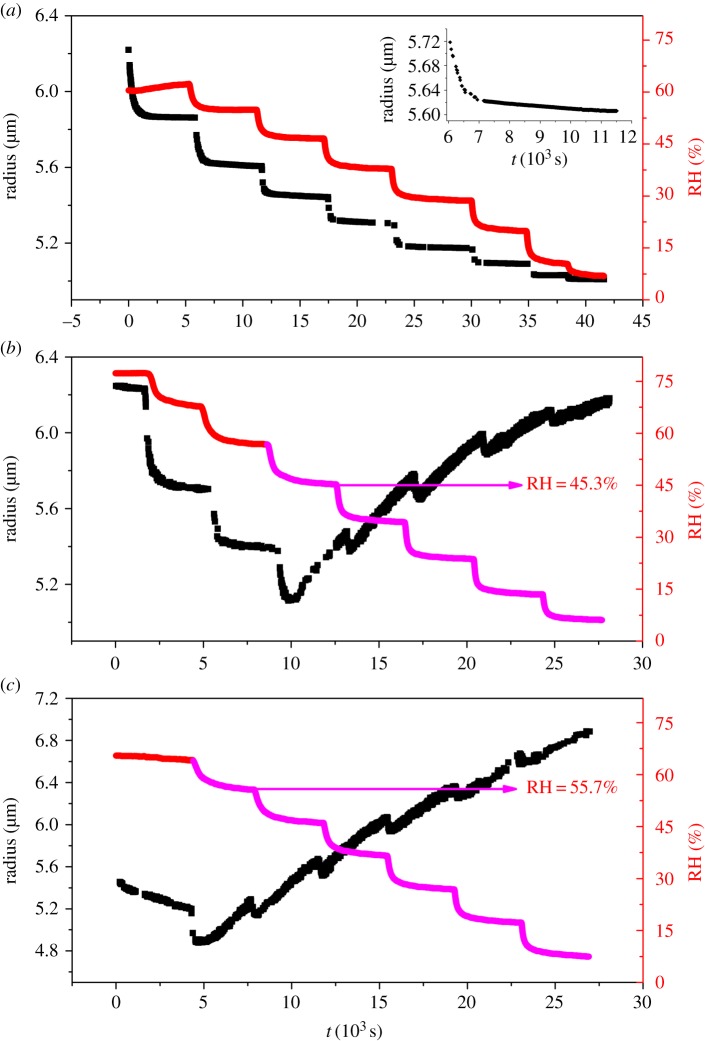
Time-dependent glycerol/NaNO_3_/H_2_O droplet size (black points) and RH (red and magenta line) with different OIRs are presented. (*a*–*c*) The molar ratios of 3 : 1, 1 : 1 and 1 : 3, respectively. The inset of figure 2*a* shows an expanded view of the steady evaporation of glycerol at a constant RH of 54.9%.

During the experiments, the RHs were varied in a sequence of steps by steadily decreasing the RH in small steps. As shown in figure 3*a*, stepwise changes in RH induce step changes in radius of glycerol/NaNO_3_/H_2_O droplet (OIR = 3 : 1), indicating that the droplet diminishes through evaporation of water to maintain an equilibrium balance in water activity that matches the decrease in RH. Once the hygroscopic response to the RH change is completed, the subsequent linear decline in particle size arises from the slow volatilization of glycerol with the much lower vapour pressure from the glycerol/NaNO_3_/H_2_O droplet, accompanied by the solvating water, as shown in the inset of figure 3*a*, where the radius declines only 18.5 nm during 4509 s at 54.9% RH. The reason should be attributed to the higher activity of glycerol in the levitated glycerol/NaNO_3_/H_2_O droplet than that in the surrounding gas phase. Therefore, the droplet shrinks at any constant RH that we investigated mainly due to the evaporation of glycerol in the levitated droplet.

The changes in radius of glycerol/NaNO_3_/H_2_O droplet (OIR = 1 : 1) display the similar trend to that of figure 3*a* when the RH declines from 77.3% to 56.8%, as shown in figure 3*b*. With the subsequent decrease in RH from 56.8%, the radius of glycerol/NaNO_3_/H_2_O droplet diminishes initially with the sudden decrease in RH at the first approximately 300 s in each step for the hygroscopic response to the RH changes. Interestingly, when the RH is constant in the following steps (from 45.3% RH to 6.1% RH), the radius increases rather than continuing to slowly decrease as in figure 3*a*. For example, the radius increases 357.6 nm during 3332 s at the constant RH of 45.3%. For the mixed glycerol/NaNO_3_/H_2_O droplet with OIR = 1 : 3 (figure 3*c*), the radius of glycerol/NaNO_3_/H_2_O droplet also decreases at the beginning of each step and then increases with invariable RH from 55.7% (increase 406.5 nm within 2933 s) to 7.5% (increase 290.6 nm within 3257 s). The main reason for the droplet shrinkage at high constant RHs is the evaporation of glycerol in the levitated mixed droplet, because of the higher activity of glycerol in the levitated droplet than that in the gas phase. On the contrary, the droplet growth at low RHs is mainly ascribed to the condensation of glycerol. The main reason for this process can be attributed to the crystallization of NaNO_3_ in the surrounding deposited glycerol/NaNO_3_/H_2_O droplets on the inner wall of the chamber because of heterogeneous nucleation of NaNO_3_. Once NaNO_3_ crystallizes in the deposited glycerol/NaNO_3_/H_2_O droplets, the activity of glycerol in the deposited mixed particles would be higher than that of the levitated mixed droplet, resulting in the evaporation of glycerol from the surrounding glycerol/NaNO_3_/H_2_O particles and condensation of the glycerol to the levitated glycerol/NaNO_3_/H_2_O droplet. Therefore, glycerol repartitioning between the crystallized particles and the levitated droplet occurs [54].

**Figure 4.**
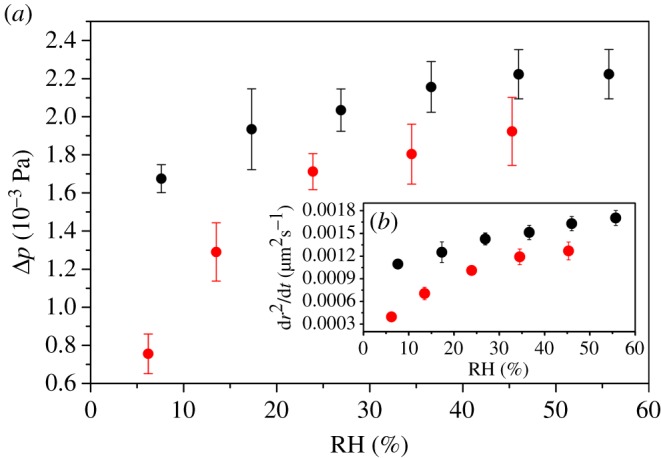
The values of Δ*p* and d*r*^2^/d*t* for the OIRs of 1 : 1 (red) and 1 : 3 (black) at different RHs after NaNO_3_ crystallized in the deposited droplets.

The radius of levitated droplet changed at constant RHs depends on the difference between the vapour pressure of the glycerol at the levitated droplet surface (*p_r_*) and the partial pressure at infinite distance (*p_∞_*); this is written as Δ*p = p_∞_* − *p_r_*. Here, *p_∞_* can be assumed as the vapour pressure of the glycerol at the deposited droplet surface surrounding the inner wall of a chamber, according to the Maxwell equation:

$$\frac{\text{d}r^{2}}{\text{d}t}=\frac{2\text{MD}}{\text{RT}\rho F}\Delta p,$$

where *M* is the molecular mass of glycerol, *D* is the diffusion coefficient of glycerol in the surrounding gas, *R* is the ideal gas constant, *T* is the temperature, *ρ* is the density of the droplet and *F* is the mass fraction of glycerol in the droplet. The diffusion constant of glycerol (8.147 × 10^−6^ m^2^ s^−1^) is estimated in nitrogen at 298.15 K using the equations of Chapman and Enskog and from Lennard-Jones potential parameters [55]. Here, in order to investigate the changes of Δ*p* after NaNO_3_ crystallizes, the values of d*r*^2^/d*t* and Δp were calculated for the OIRs with 1 : 1 and 1 : 3 at different constant RHs after NaNO_3_ crystallized in the deposited droplets, and they are presented in figure 4*a*,*b*, respectively. For the same RHs, both d*r*^2^*/*d*t* and Δ*p* increase with decreasing OIR. The values of *p_∞_* can be assumed the same value after the NaNO_3_ crystallized at the fixed RH, so the main reason for these phenomenon can be ascribed to the vapour pressure values of the glycerol at the levitated droplet surface decreasing with increasing ratio of NaNO_3_. Furthermore, for the same ratio of NaNO_3_, the value of Δ*p* also decreases with decreasing RH. The reason can be attributed to the vapour pressure of the glycerol at the levitated droplet surface increasing when the RH decreases [30]. Moreover, both the values of d*r*^2^*/*d*t* and Δ*p* are positive, indicating that the vapour pressure of the glycerol at the levitated droplet surface is lower than that at the deposited droplets surface, which results in the evaporation of glycerol from the deposited droplets to the levitated droplet. Thus, the repartitioning of glycerol occurs, which is consistent with the above results.

**Figure 5.**
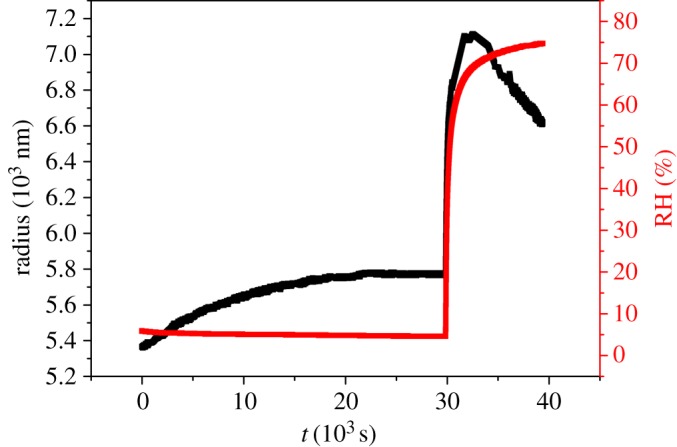
Time dependence in the levitated glycerol/NaNO_3_/H_2_O droplet size (black points) and RH (red line) with OIR 1 : 1.

Figure 5 shows the changes in radius of glycerol/NaNO_3_/H_2_O droplet (OIR = 1 : 1) with constant RH at 5% about 30 000 s and then up to 74% RH. The radius of the droplet increases gradually (411.9 nm) until the time reaches about 25 000 s, and then is invariable at the constant RH, showing that the equilibrium of glycerol activity between the levitated droplet and the gas phase is reached at this period. After that, the RH experiences a rapid upwards trend from 5% to 74%, for the hygroscopic response to the RH change, the radius of the levitated glycerol/NaNO_3_/H_2_O droplet also increases rapidly. When the RH maintains at approximately 74%, the radius starts to decrease significantly (reducing 497.9 nm within 6888 s). The reason could be ascribed to the deliquescence of NaNO_3_ in the surrounding deposited glycerol/NaNO_3_/H_2_O particles at 74% RH. With the deliquescence of NaNO_3_, the activity of glycerol in the deposited droplets should be much lower than that of the levitated droplet, leading to evaporation of glycerol from the levitated glycerol/NaNO_3_/H_2_O droplet, thus the pronounced droplet shrinkage occurs at 74% RH. In order to further illustrate the reason for the sharp decrease of the levitated droplet radius, we calculate the glycerol to NaNO_3_ molar ratio which is 5.5 : 1 at 5% RH according to the refractive index of the droplet (1.461) when the equilibrium of glycerol activity between the levitated droplet and the gas phase is reached, which means the OIR is increased from 1 : 1 to 5.5 : 1 owing to the transfer of glycerol from the deposited droplets to the levitated droplet. The OIR at 74% RH is also calculated based on the refractive index of the droplet (1.396), and the value of it is 5.3 : 1 which is nearly close to the value of the levitated droplet before the increase in RH. After long time (25 000 s) of glycerol evaporation in the deposited droplet at the low RH (5% RH), when RH is rapidly increased, the NaNO_3_ crystal deliquesces, leading to the much lower concentration of glycerol in the deposited droplets than that of the levitated droplet, thus the radius of the levitated droplet decreases sharply.

### 3.2. The process of glycerol evaporation

**Figure 6.**
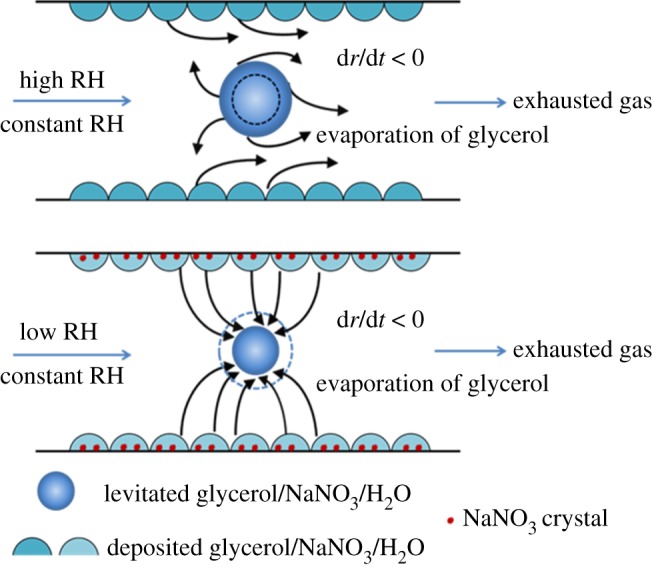
The schematic diagram of the evaporation of glycerol in the glycerol/NaNO_3_/H_2_O droplets.

Figure 6 illustrates the schematic diagram of the phenomenon discussed in §3.1. At high RHs, the glycerol evaporates from the levitated and deposited glycerol/NaNO_3_/H_2_O droplets to the gas phase, because of the higher activity of glycerol in the mixed droplets than that in the gas phase, leading to the droplets shrinkage with increasing time at constant RH blowing. However, as to the low RHs, owing to the efflorescence of the NaNO_3_ in the surrounding deposited droplets, the activity of glycerol in the surrounding deposited droplets is higher than that in the levitated droplet, resulting in repartitioning of glycerol from the surrounding deposited droplets to the levitated droplet, thus the radius of the levitated droplet increases gradually. From the above-mentioned analysis, we can conclude that the difference of the glycerol activity between the levitated glycerol/NaNO_3_/H_2_O droplet and the surrounding efflorescence glycerol/NaNO_3_/H_2_O particles leads to the repartitioning of glycerol between them.

According to the above discussions, in the figure 3, the mixed droplet (OIR = 3 : 1) shrinkage at any constant RHs indicates that no efflorescence process occurs for both the levitated and surrounding deposited glycerol/NaNO_3_/H_2_O droplets even though the RH decreases close to 0%. However, as to the mixed droplets with OIRs of 1 : 1 and 1 : 3, the levitated droplet growth occurs except for the droplet shrinkage, indicating that the efflorescence process of the deposited droplets with OIRs of 1 : 1 and 1 : 3 begins at 45.3% and 55.7% RH, respectively. The efflorescence RH for the deposited glycerol/NaNO_3_/H_2_O droplets decreases with increasing OIR, which is consistent with our previous studies [47,48]. On the other hand, the levitated droplet does not effloresce in the whole RH range because only homogeneous nucleation of NaNO_3_ exists in the levitated droplet, in which the homogeneous nucleation rate is much lower than the heterogeneous nucleation rate that occurred in the deposited droplets at the same RH.

## 4. Conclusion

For the first time, the repartitioning of glycerol between a levitated glycerol/NaNO_3_/H_2_O droplet and surrounding deposited glycerol/NaNO_3_/H_2_O droplets was investigated via optical tweezers coupled with cavity-enhanced Raman spectroscopy. For the high OIR with 3 : 1, no NaNO_3_ crystallization for both levitated and deposited droplets occurs even at RH close to 0%. With blowing of constant RH, the radius of the levitated droplet is slowly decreasing, corresponding to evaporation of glycerol from the levitated droplet. For the mixed droplet with lower OIRs, repartitioning of glycerol occurs via gas phase exchange of glycerol between the externally mixed of the deposited glycerol/NaNO_3_/H_2_O droplets and the levitated glycerol/NaNO_3_/H_2_O droplet when blowing with constant RH lower than 45.3% for 1 : 1 or 55.7% for 1 : 3. Because of the crystallization of NaNO_3_ in the deposited droplets on the inner wall of the chamber, the activity of glycerol for the deposited ones is higher than that of the levitated one which always remains droplet without NaNO_3_ crystallization, resulting in the transfer of glycerol from the deposited ones to the levitated one.

Through this work, we show that the optical tweezers coupled with cavity-enhanced Raman spectroscopy technology is capable of investigating not only hygroscopic and volatile behaviour of optical levitated single SVOC/inorganic salt aerosol droplet but also the repartitioning of SVOC between the levitated SVOC/inorganic salt droplet and the surrounding SVOC/inorganic salt droplets deposited on the inner wall of a chamber. In the atmosphere, with changing climate conditions, such as decreasing RH, the internally mixed SVOC/inorganic salt aerosol droplets could experience efflorescence process or enter into supersaturated state when RH is lower than crystallization RH of inorganic salt. If there is no seed in an internally mixed SVOC/inorganic salt aerosol droplet, similar to the levitated glycerol/NaNO_3_ droplet in this work, SVOC would suppress the homogeneous nucleation rate of inorganic salt and thus the aerosol would exist in liquid state even at low RH. At the same low RH, however, an internally mixed SVOC/inorganic salt aerosol droplet containing seed, similar to that deposited on the wall in this work, would experience crystallization of the inorganic salt because of heterogeneous nucleation. Thus there would be a different vapour pressure for SVOC at the particle surface of these two kinds of aerosols. It means that the phase states of externally mixed SVOC/inorganic salt particles could be different in the same climate condition (such as at the same low RH). This can result in the vapour pressures of SVOC at particle surface with different phase state being different. Thus, the SVOC would transfer from the particle with high surface vapour pressures to the particle with low surface vapour pressures of SVOC. The results obtained herein will be of important significance in understanding repartitioning of SVOCs between the externally mixed particles with different phase states.

## Supplementary Material

GN.xlsx file containing all the original data

## Data accessibility

All data were created using the Excel file (GN.xlsx) supplied as the electronic supplementary material.
